# Elevated blood pressure in adolescent girls: correlation to body size and composition

**DOI:** 10.1186/s12889-016-2717-6

**Published:** 2016-01-26

**Authors:** Ashley L. Devonshire, Erin R. Hager, Maureen M. Black, Marie Diener-West, Nicholas Tilton, Soren Snitker

**Affiliations:** 1 Department of Pediatrics, University of Maryland School of Medicine, Baltimore, Maryland USA; 2 Department of Biostatistics, Johns Hopkins Bloomberg School of Public Health, Baltimore, Maryland USA; 3 Department of Pediatrics, Northwestern University Feinberg School of Medicine, Chicago, Illinois USA; 4 Department of Medicine, University of Maryland School of Medicine, 660 West Lombard Street, Rm. 598-B, 21201 Baltimore, Maryland USA

**Keywords:** Hypertension, Obesity, Anthropometry, Waist-to-height ratio, Fat-free mass

## Abstract

**Background:**

To improve understanding of the pathophysiology of hypertension in adolescents and pave the way for risk stratification, studies have sought to determine the correlates of blood pressure (BP). Inconsistencies in dependent and independent variables have resulted in an elusive consensus. The aim of this report is to examine an inclusive array of correlates of BP, as a continuous (systolic and diastolic BP) and a dichotomous variable.

**Methods:**

Subjects were a school-based sample of 730 urban, mostly African American, non-referred 6th and 7th grade girls. To find independent correlates of SBP/DBP, we used a stepwise model selection method based on the Schwarz Bayesian Information Criterion, enabling selection of a parsimonious model among highly correlated covariates. Candidate variables were: age, stature, heart rate, pubertal development, BMI, BMI *z*-score, waist circumference, waist-to-height ratio (WHtR), body surface area, fat mass (by bioelectrical impedance analysis), fat-free mass (FFM), percentage of body fat, and presence of overweight/obesity.

**Results:**

The best-fitting models for DBP and SBP (considered separately) included fat-free mass, heart rate and, in the case of SBP, stature. The best-fitting model for high-normal/elevated blood pressure (H-N/EBP) included WHtR with no independent relation of any other variable. The prevalence of H-N/EBP tripled between a WHtR of 0.5 and 0.7.

**Conclusions:**

The easily obtained and calculated WHtR is the strongest correlate of elevated blood pressure among available variables and is a prime candidate for longitudinal studies of predictors of the development of hypertension.

**Trial registration:**

ClinicalTrials.gov Identifier, NCT00746083

## Background

Hypertension (HTN), i.e., elevated blood pressure, is one of the most important risk factors for cardiovascular disease. To better understand its pathophysiology and identify risk markers, i.e., variables that identify individuals at risk for developing HTN, an obvious starting point is to identify the cross-sectional correlates of HTN. Although several studies have been published in adolescents, a consensus is elusive because studies vary in their choice of dependent variable (e.g., the continuous variable *blood pressure* or the dichotomous variable *hypertension*) and independent variables.

With regard to blood pressure as a continuous variable, studies in youths have found that blood pressure (BP) increases with stature, BMI, and heart rate (HR) [1, 2]. Other studies have reported that indices of central obesity – such as waist circumference (WC) – are correlated with HTN in adolescents [3, 4], in agreement with the notion that central obesity, i.e., fat deposition inside the abdominal cavity, contributes to the pathophysiology of HTN [5]. However, it is controversial whether models including both BMI and a measure of central obesity are superior to models including only one of these.

Another important question is whether age and pubertal stage, both of which are correlated with BP [1, 6], remain correlated with BP when controlling for their effects on stature and fat accretion. Race is another potential correlate that may possibly be broken down into more proximate factors.

To address this knowledge deficit, this study aims to evaluate, in a non-referred sample of adolescent girls, which subset of variables provides the best model to explain (1) inter-individual variation in BP (separately for SBP and DBP) and (2) the presence of pathologically high BP. Extending beyond the limited number of variables considered in other studies, we included BMI-for-age, other anthropometric indices, body composition, HR, stature, age, pubertal development, and race. Notably, we also included the variable fat-free mass (FFM), which is seldom considered by investigators. Because practically all the examined independent variables are correlated, we used a model selection method based on the Schwarz Bayesian Information Criterion [7]. This method enables selection of a parsimonious model from a very large number of highly correlated effects [8].

The participants were primarily African American adolescent girls in a Mid-Atlantic, large, urban school district serving mostly low-income communities. The relative homogeneity of the sample confers a high degree of internal validity, i.e., findings are less likely to be explained by non-measured confounders than if the sample had been drawn from a broader geographical area and ethnic mix. Moreover, because obesity is related to socioeconomic factors [9] this group merits particular attention.

## Methods

We used baseline observations from a randomized, controlled trial of girls attending grades 6 and 7 in 22 Baltimore City schools. Girls were invited to participate through mailings and in person during lunch breaks and at school-sponsored events. The randomized element of the trial was assignment to either (1) nutrition and physical activity or (2) bullying prevention. Data were collected prior to randomization. Exclusion criteria were language barriers, competing time commitments, and medical conditions that would interfere with any of the 2 interventions or pose an increased risk. All participants provided informed assent and their primary caregivers provided informed consent. This study was approved by the U of Maryland, Professional Schools IRB and the Baltimore City Public Schools IRB.

### Measures

By self-report, participants selected among 5 racial categories with the option of selecting multiple categories as well as an “Other,” write-in category.

Anthropometric measurements were taken by trained research staff. Height was measured to the nearest 0.1 centimeter with a portable stadiometer (Shorr Productions, Olney, Maryland) in triplicate and averaged. Waist circumference (WC) was measured at the level of the umbilicus with a tape measure to the nearest 0.1 centimeter in triplicate and averaged. WHtR was calculated as waist circumference divided by height. Body weight, fat mass, fat-free mass (all to the nearest 0.1 kg), and percent body fat (to the nearest 0.1 %) were assessed using a body composition analyzer (Tanita TBF-410, Arlington Heights, IL), which combines a traditional scale with bioelectrical impedance analysis (BIA). Participants removed socks, jewelry and pocket contents prior to stepping on the device. BIA measurements were performed twice; if weight or percent body fat differed, a third measurement was taken and the average calculated.

BMI was calculated for each participant as weight divided by height squared (kg/m^2^). BMI *z*-scores and percentiles were computed and participants were categorized as normal weight (<85^th^ percentile) or overweight/obese (≥85^th^ percentile) based on the Centers for Disease Control and Prevention growth charts [10]. Body surface area (BSA) was calculated according to Du Bois’s formula [11] as 0.007184 · weight^0.425^ · height^0.725^.

Pubertal status was assessed using a validated self-assessment questionnaire with drawings of the Tanner puberty stages [12]. Illustrations depicted breast development and pubic hair distribution. Scores on the scales for the two characteristics, which were correlated (*r* = 0.499; *p* <0.001), were averaged into a single puberty score with a theoretical and observed range from 1-5.

BP and HR were measured using a professional, digital oscillometric BP monitor (Omron, HEM-907XL, Bannockburn, IL), validated within ± 3 mmHg or 2 % for BP and within ± 5 % of the reading for HR. Cuff size was selected based on measured arm circumference. BP was measured after a 3-minute rest, with the participant in a seated position and her right arm in an extended position resting at heart level with palm facing up. After the first measurement, the participant was instructed to raise her arm in the air and to clench and release her hand three times and, approximately one minute later, a second measurement was taken. If the first two BP measurements differed by more than 10 %, a third measurement was taken. The two closest measurements were used for computation of an average SBP and average DBP. In rare cases (*n* = 16), BP measurements were repeated 4, 5, or 6 times. Average HR was calculated in a similar fashion.

Age-, gender-, and height- specific SBP and DBP *z*-scores and percentiles according to national standards were computed as described in the *Fourth Report on the Diagnosis, Evaluation, and Treatment of High Blood Pressure in Children and Adolescents* from the National High Blood Pressure Education Program [13]. Individuals were categorized into the following BP groups: a) normal BP (both SBP and DBP <90^th^ percentile) and b) high-normal/elevated BP (H-N/EBP, either SBP or DBP ≥90^th^ percentile) [13].

### Statistical analysis

Data were analyzed using SAS Version 9.2 (SAS Institute; Cary, NC). The first part of the analysis identified the correlates of DBP and SBP. A Pearson’s correlation matrix was calculated for all pairs of dependent and independent variables. To establish the correlates of DBP and SBP in the framework of general linear models, independent variable selection was performed in a stepwise manner according to the Schwarz Bayesian Information Criterion (SBC) as implemented in SAS PROC GLMSELECT [8]. Decisions about what variables to add or drop at any step and when to terminate the selection were based on the SBC [8]. In addition to height, HR, puberty score, and race, the following measures of body size were considered: BMI, BMI *z*-score, BMI category (normal v. overweight/obese), WC, WHtR, BSA, body weight, percent body fat, fat mass, and FFM.

To account for possible intra-school clustering, the selected models were examined further in SAS PROC GENMOD, which accounts for clustered data using the generalized estimating equation.

The second part of the analysis focused on the statistical correlates of HTN. Subjects were characterized according to BP category. Group differences were examined by *t*-tests for continuous and *X* ^2^ tests for categorical variables. Initial variable selection was performed by stepwise logistic regression according to SAS PROC LOGISTIC. The selected variables were further examined in PROC GENMOD to account for possible intra-school clustering while specifying the appropriate distribution for logistic regression. Log odds ratio estimates were converted to odds ratios by exponential transformation. Prevalence was calculated as 1/(1 + odds ratio).

## Results

Out of 1840 girls in grades 6 and 7 in the participating schools, 789 (43 %) were enrolled. Less than 5 % of girls seeking enrollment were excluded for any reason, including medical conditions, language barriers, and competing time commitments. By self-identified racial/ethnic category 701 girls (90.3 %) were African American/Black, including 29 girls who additionally selected one or more other categories, 29 were solely Caucasian/White, 26 were solely American Indian/Native American, 16 were solely Hispanic, and 4 were solely Asian. Race/ethnic category was coded as missing for girls who declined to provide this information (*n* = 13).

Due to time and staffing constraints, BP and HR measurements were omitted on 59 participants. There was no significant difference (*p* <0.05) in the distribution of any subject characteristic according to whether BP measurements were or were not performed (data not shown), leading us to conclude that BP data were missing at random. All further analyses were conducted on the 730 participants with complete BP and HR data; their characteristics are presented in Table 1. The age range at examination was 10.0 – 14.7 years. Mean (SD) SBP/DBP was 104.4 (9.6)/59.1 (7.8) mmHg.

**Table 1:** Characteristics of the 730 participating girls

	All participants (*n* = 730)	BP < 90^th^ percentile (*n* = 690)	BP ≥90^th^ percentile (*n* = 40)	p*
Age (years)	12.1 (0.7)	12.1 (0.7)	12.2 (0.8)	0.44
Puberty stage	3.2 (1.0)	3.2 (0.9)	3.2 (1.0)	0.81
Race = AA^a^	91 %	91 %	95 %	0.34
HR (min^-1^)	81.8 (9.9)	81.6 (9.9)	85.0 (8.8)	0.03
Weight (kg)	57.1 (16.9)	56.6 (16.6)	65.6 (19.9)	0.008
Height (cm)	155.4 (7.4)	155.4 (7.4)	155.5 (7.4)	0.97
BMI	23.5 (6.0)	23.3 (5.9)	27.0 (7.2)	0.0001
BMI *z*-score	1.00 (1.04)	0.97 (1.03)	1.5 (1.03)	0.003
BMI ≥ 85^th^ percentile	51 %	49 %	73 %	0.007
Waist circumference (cm)	77.7 (14.2)	77.2 (14.0)	85.7 (15.8)	0.002
Waist/height ratio	0.500 (0.087)	0.497 (0.085)	0.551 (0.100)	0.002
Fat Mass (kg)	18.4 (11.2)	18.1 (11.1)	24.3 (12.6)	0.04
Body surface area (m^2^)	1.54 (0.22)	1.54 (0.22)	1.64 (0.23)	0.01
Fat-free mass (kg)	38.7 (6.3)	38.5 (6.2)	41.4 (7.8)	0.006
Body fat (%)	29.7 (9.9)	29.4 (9.9)	34.6 (9.4)	0.002

Values are mean ± SD or percentages. *Difference between BP < 90th percentile and BP ≥ 90th percentile determined by *t*-test or *X* ^2^ test.^a^AA, African American

### Correlates of DBP and SBP

The correlates of DBP and SBP were examined separately. Table 2 shows selected pairwise Pearson correlation analyses. Both DBP and SBP were correlated to age, puberty score, heart rate, height, and all measures of body size (BMI *z*-score, BMI, WC, WHtR, BSA, body weight, percent body fat, fat mass, and fat-free mass) with correlation coefficients ranging from 0.08 – 0.30. In addition, all measures of body size were strongly related to each other with correlation coefficients ranging from 0.66 – 0.96.

**Table 2:** Correlation matrix of selected variables (duplicates omitted)

	DBP	SBP	BMI *z*-score	BMI	WC	WHtR	%fat	FFM
Age	0.09**	0.12***	−0.03	0.09*	0.10**	0.01	0.11**	0.26***
Puberty stage	0.08*	0.09*	0.26***	0.23***	0.21***	0.12**	0.27***	0.36***
Heart rate	0.26***	0.10**	−0.04	−0.00	0.03	0.05	−0.03	−0.03
Height	0.16***	0.25***	0.21***	0.23***	0.31***	0.05	0.35***	0.63***
Weight	0.30***	0.26***	0.85***	0.95***	0.92***	0.82***	0.88***	0.93***
BMI *z*-score	0.27***	0.21***	-	-	-	-	-	-
BMI	0.30***	0.21***	0.91***	-	-	-	-	-
WC	0.29***	0.21***	0.86***	0.94***	-	-	-	-
WHtR	0.26***	0.15***	0.85***	0.92***	0.96***	-	-	-
BSA	0.28***	0.29***	0.79***	0.86***	0.86***	0.71***	0.85***	0.95***
%fat	0.26***	0.19***	0.89***	0.89***	0.88***	0.83***	-	-
FM	0.29***	0.22***	0.85***	0.96***	0.93***	0.86***	0.93***	0.84***
FFM	0.30***	0.30***	0.75***	0.82***	0.80***	0.66***	0.69***	-

*, *p* ≤0.05; **, *p* ≤0.01; ***, *p* ≤0.001

The variable selection for DBP involved 2 steps, adding FFM (*t* = 8.89) and HR (*t* = 7.77), respectively. For SBP the variable selection involved 3 steps, adding FFM (*t* = 5.11), HR (*t* = 3.25), and height (*t* = 2.66), respectively.

The validity of the selected models was then examined using the generalized estimating equation to take intra-school clustering into account. *β*-coefficients and *p*-values are shown in Table 3. DBP increased by 0.35 mmHg for each kg increase in FFM and by 0.20 mmHg for each bpm increase in HR. SBP increased by 0.32 mmHg for each kg increase in FFM, by 0.01 mmHg for each bpm increase in HR, and by 0.16 mmHg for each cm increase in height.

**Table 3:** Parameters from the generalized estimating equation for the final, selected models explaining DBP (top panel) and SBP (bottom panel)

*Dependent variable DBP*				
Independent variable	*β*	SE	*Z*	*p*
Fat-free mass (kg)	0.352	0.040	8.72	<0.0001
Heart rate (min^-1^)	0.201	0.020	10.29	<0.0001
*Dependent variable SBP*				
Independent variable	*β*	SE	*Z*	*p*
Fat-free mass (kg)	0.317	0.060	5.27	<0.0001
Heart rate (min^-1^)	0.091	0.029	3.45	0.0006
Height (cm)	0.159	0.041	3.84	0.0001

### Correlates of high-normal/elevated BP

Subject characteristics according to BP group are given in Table 1. The presence of H-N/EBP (a BP ≥ the 90th percentile) was correlated to all measures of body size except height, but not to age, race, or puberty score.

**Fig. 1:**
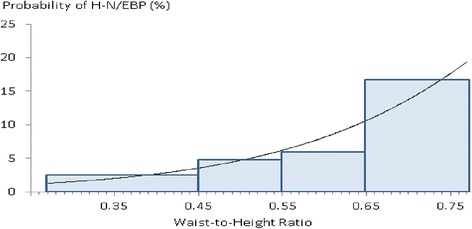
The curve is the prevalence estimate of H-N/EBP (high-normal/elevated blood pressure) in 730 adolescent girls as a function of waist-to-height ratio according to the generalized estimation equation (*p* <0.0001). Columns are observed prevalence stratified according to waist-to-height ratio (*N* = 257, 284, 141, and 48 respectively)

The stepwise, logistic regression variable selection procedure with H-N/EBP as the dependent variable selected WHtR as the sole independent correlate of H-N/EBP. The correlation with WHtR was confirmed using the generalized estimating equation to take intra-school clustering into account. The log odds ratio parameter estimate from the generalized estimating equation was 5.89 for WHtR (*p* <0.0001). To visualize this parameter, the log odds ratio was converted to a prevalence estimate as shown in Fig. 1 along with observed prevalences. Neither age, puberty score, race, nor any body size variable entered the model.

## Discussion

In multivariable analyses the variable that explained the greatest variability in DBP and SBP was FFM. Most studies of the relation between body composition and BP consider only measures of fatness even though FFM is always available whenever fat mass or percentage of body fat is assessed. However, in this and the few other studies [14, 15] where FFM or lean body mass has been included, it is consistently the strongest correlate of BP. Paraphrasing Daniels et al. [14], who made the same observation, this is a biologically plausible finding because mean arterial pressure is the product of cardiac output × total peripheral resistance, cardiac output is likely to be regulated by the body’s metabolic demand, and metabolic demand is much lower in fat than in lean body mass. This theory would also explain why BSA, which is also highly correlated to whole-body metabolic rate, explains almost as much of the variation in DBP/SBP as does FFM in this and other studies [14].

Following FFM, the variable that explained the greatest inter-individual variability in BP was HR. HR was not correlated to FFM or any other measure of body size, making HR and FFM independent correlates of BP. It is well-known that HR and BP are correlated in rested individuals [14, 15]. Incidentally, HR and BP increase in tandem during acute exercise, suggesting that the correlation in supposedly rested individuals may in part be explained by some of these not having rested for an adequate amount of time. This theory might help explain why adjustment for HR tends to strengthen the relation between body size and BP, and would be testable by measuring vital signs in a group of people after variable periods of rest.

Height was independently associated with SBP and has – like FFM and HR – previously been associated with BP in both adults and children. This finding has been attributed to the necessity of a higher BP to secure adequate cerebral perfusion in the presence of the higher hydrostatic pressure generated between the brain and the heart in a taller person. Consistent with reports of others [2], we did not detect any independent effect of age when body size was included in the model. Likewise, puberty score was not independently associated with BP despite the fact that the sample represented the whole range of pubertal development with values from 1 to 5 and a mean score of 3.2. These findings suggest that effects of age and puberty on BP in adolescents are mostly, if not exclusively, accounted for by changes in body size with development.

We did not find an independent effect of race on BP. While this lack of evidence may be due to limited statistical power as a result of the small cell sizes for all groups but African Americans it is consistent with a study of 98 African Americans and 103 Caucasians [14].

In contrast to our analysis of blood pressure as a continuous variable, the stepwise selection procedure indicated that WHtR was the sole independent correlate of H-N/EBP. As shown in Fig. 1, a girl with a WHtR of 0.7 had as much as a 3-fold increased risk of H-N/EBP compared to the average participant with a WHtR of 0.5. For an average height of 155 cm, a 0.2 difference in WHtR corresponds to a difference in waist circumference of 155 cm · 0.2 = 31 cm.

WHtR was first used as a measure of central obesity in the Framingham Heart Study [16]. For use as a pediatric risk marker, WHtR has some advantages compared to other anthropometric measures. First, it is practically unrelated to age as demonstrated in the present study and by others [3, 4]. Second, the same cutoff (e.g., 0.50) may indicate the same relative risk in both children and adults and in both genders [4], obviating the need to calculate age- and sex- specific *z*-scores or percentiles.

The correlation between WHtR and H-N/EBP is biologically plausible because the adverse consequences of excess weight are thought to be explained by adipokines [17] such as leptin, adiponectin, components of the renin-angiotensin system, and inflammatory factors (e.g., TNF-α, IL-6, and CRP). Fat is stored in several depots, each of which is associated with its own unique adipokine profile and appurtenant risk [17]. While visceral adipose tissue (VAT), i.e., fat deposited within the abdominal cavity, seems particularly insidious, its volume being strongly correlated to the presence of type 2 diabetes, dyslipidemia, and atherosclerosis [5], some evidence suggests that subcutaneous fat on the extremities may have neutral or even health-protective effects [18]. Magnetic resonance studies have shown that the strongest anthropometric correlate of VAT area in obese girls aged 12-18 is WHtR [19], with a correlation coefficient of 0.82. In light of this evidence, it is not surprising that WHtR emerged as the strongest correlate of H-N/EBP in the present study and as the strongest correlate of CVD risk markers in other studies [20, 21], although a more accurate, imaging-based measure of VAT volume might have performed even better.

In summary, in this population of adolescent girls of whom 5.5 % met criteria for H-N/EBP we found conflicting statistical findings depending on whether our dependent variable was BP (continuous variable) or pathologically elevated blood pressure (dichotomous variable). As a synthesis of our data and the mechanistic studies of others, we posit that in generally healthy youths with a modest enrichment for HTN, the independent correlates of BP studied as a continuous variable mostly reflect inter-individual variation in BP within the normotensive range, which is explained by physiological adaptation to linear growth and expansion of lean tissue rather than visceral fat deposition. On the other hand, the correlation between WHtR and elevated BP (as a dichotomous variable) reflects pathophysiological processes revolving around VAT deposition, superimposed on the mechanisms involving lean tissue.

Consistent with our observation that it is visceral fat accumulation, not FFM, that distinguishes H-N/EBP, physical activity interventions do not increase risk of HTN although they increase lean mass. In fact, in controlled studies, assignment to an exercise program has been successful in reducing BP in obese children [22–24].

In analogy to other studies of children and adults [3], our data did not support the relevance of a model including a general body size measure (e.g., BMI *z*-score) and a measure of central obesity (e.g., WC).

### Strengths and limitations

Like most of the work on which current knowledge is based [3, 4, 14, 15, 20, 21], our study is cross-sectional, prohibiting us from making causal inferences. Indeed, a large FFM and a high stature could be secondary to a high blood pressure, or they could be separate effects of an underlying unmeasured factor.

Several minor limitations exist. (1) We have no way of gauging to which extent the 43 % of the 6^th^ and 7^th^ grade girls that enrolled are representative of the 22 participating schools. Less than 5 % of girls who sought enrollment were excluded for any reason, medical or practical. (2) We did not collect information on antihypertensive use, albeit rare in this age group. (3) BP was measured by oscillometry. Although this method correlates well with intra-arterial BP measurements, auscultation is the preferred method [25]. (4) Sexual maturation staging was not based on objective observation; however, it was based on a validated measure and its correlation to height and adiposity provides convergent validity to the subjective reports. (5) The assessment of body composition was done within the schools with the BIA method rather than the more accurate dual-energy X-ray absorptiometry, which would have required a laboratory visit.

These 5 limitations are minor because the main purpose of this investigation was variable selection in the context of associations between variables, not the exact assessment of population estimates.

The report adds to current knowledge because as far as we are aware no other pediatric study of this topic examines as independent variables both FFM and WHtR and as dependent variables both the continuous variables of SPB and DBP and the dichotomous variable of elevated BP. This comprehensive analytical approach promotes novel insights and confirms that FFM, although seldom considered by investigators, is reproducibly the strongest anthropometric correlate of BP [14, 15].

## Conclusions

We found that (1) inter-individual variation in DBP and SBP was best explained by FFM and HR (and height in the case of SBP), and (2) the presence of elevated blood pressure, defined as SBP or DBP at or above the 90^th^ percentile, was best explained by WHtR, tripling in prevalence for those with a WHtR of 0.7 as compared to 0.5. Our findings suggest the relevance of longitudinal investigations to determine the utility of using WHtR – alone or with other biomarkers – to risk stratify adolescents for HTN.

## Abbreviations

BIA: Bioelectrical impedance analysis
BMI: Body mass index
BP: Blood pressure
SBP: Systolic blood pressure
DBP: Diastolic blood pressure
HR: Heart rate
WC: Waist circumference
WHtR: Waist-to-height ratio
VAT: Visceral adipose tissue
